# The role of Pediatric Surgery in childhood cancer

**DOI:** 10.1590/1806-9282.2024S110

**Published:** 2024-06-07

**Authors:** Vilani Kremer, Wilson Elias de Oliveira

**Affiliations:** 1 Erastinho Pediatric Oncology Hospital – Curitiba (PR), Brazil.; 2 Universidade Federal do Paraná, Clinical Hospital – Curitiba (PR), Brazil.; 3 Barretos Cancer Children's Hospital – Barretos (SP), Brazil.; 4 Medical School, Faculty of Health Sciences of Barretos "Dr. Paulo Prata" – Barretos (SP), Brazil.

## INTRODUCTION

Childhood cancer, a significant cause of morbidity and mortality among children worldwide, is the focal point of this article. With an estimated 8,460 cases diagnosed in Brazil in 2022, childhood cancer represents only 2% of all cancer cases but is a leading cause of death in children aged 1–14 years^1,2^. The incidence rate is 16.2 per 100,000 children aged below 15 years, featuring a bimodal distribution in pediatric tumors, peaking before the age of 2 years and again in adolescents and young adults. Survivors face increased risks of chronic health conditions, second malignancies, and reproductive health implications^2^.

Pediatric cancer differs from adult cancer in common types and treatment approaches. Central nervous system malignancies, neuroblastoma, acute myeloid leukemia, Wilms tumor, and retinoblastoma are more prevalent in younger children, while acute lymphoblastic leukemia, Hodgkin lymphoma, osteosarcoma, and Ewing sarcoma are common in older children^1,2^. Advances in treatment, including clinical research integration and pediatric cooperative groups, have enhanced therapy effectiveness. The focus on minimizing therapy's late effects, like infertility, led to oncofertility options in pediatric cancer treatment^3^.

Childhood cancer survival and prognosis have improved significantly. Collaborative efforts by pediatric oncology consortia and risk stratification algorithms have optimized therapy, increasing overall survival rates to 84%^4^. While the incidence remains flat, death rates have declined by 66%, with the 5-year relative survival rate improving from 58 to 83% for children and 68 to 84% for adolescents. However, certain pediatric solid tumors remain resistant to these improvements^5,6^.

Pediatric surgery plays a pivotal role in diagnosing, staging, and treating childhood cancer. Surgical resection, often the primary treatment modality for solid tumors, affects outcomes and quality of life. Pediatric surgeons also manage complications and provide supportive care, including central venous catheter insertions and symptom palliation^7^. This field demands specialized training and close collaboration with other disciplines such as oncology and pathology. The role of pediatric surgeons extends beyond surgery for solid tumors; they follow up with oncology patients, perform biopsies and catheter implants, and manage complications from treatments like neutropenic colitis. Tailoring surgical approaches to each patient's needs, considering cancer type, stage, age, and health, is crucial for optimal outcomes. Research in pediatric surgical oncology is key to developing new techniques and improving survival rates and quality of life for children with cancer^7–12^.

Pediatric surgeons are often the first point of contact for children diagnosed with tumors, building vital therapeutic relationships and guiding families through the treatment process^9,12^. Their expertise in performing biopsies, central line placements, and fertility preservation procedures is crucial for timely diagnosis and treatment^3,8,12^. As part of multidisciplinary teams, they bring anatomical knowledge and surgical expertise, performing a range of surgeries from tumor resections to complex reconstructive procedures^4^. Their understanding of children's unique needs helps in implementing appropriate treatments while minimizing complications. Despite their critical role, access to pediatric surgical oncology training is limited globally, especially in low- and middle-income countries. This highlights the need for further research and education to develop a global workforce capable of providing high-quality pediatric cancer care^7,13^. The role of pediatric surgery in childhood cancer outcomes is the focus of this article, which aims to provide an overview of the state-of-the-art and the future directions of this field.

## METHODS

We conducted a comprehensive, nonsystematic review of the literature to investigate the role of pediatric surgery in childhood cancer outcomes. Four major bibliographic databases were searched: PubMed, EMBASE, LILACS, and Web of Science. A combination of controlled vocabulary terms [Medical Subject Headings (MeSH) and Emtree terms] and keywords was employed, including "pediatric surgery" OR "surgical oncology" AND "childhood cancer" OR "pediatric cancer" AND "cancer outcome" OR "survival rate" OR "complication" OR "quality of life." Our search was restricted to articles published in English, Spanish, or Portuguese within the past 10 years, focusing on studies involving children aged 0–18 years diagnosed with childhood cancers. Initial screening involved reviewing titles and abstracts to identify relevant articles. Subsequently, full-text evaluation was conducted on selected articles, focusing on those that explicitly addressed the impact of pediatric surgery on outcome measures. Reference lists of retrieved articles were also scanned to identify additional relevant publications. Gray literature was excluded from the review.

## RESULTS AND DISCUSSION

Over time, the role of the pediatric surgeon in childhood cancer treatment has evolved significantly, becoming crucial in modern oncology due to various factors^12,14,15^. This evolution is largely attributed to the specialization and continuous training of pediatric surgeons, as highlighted by Alaish et al.^10^, who associated participation in specialized training programs with improvements in technical skills, operative time, and patient-centered performance. Moreover, the implementation of enhanced recovery programs in pediatric oncology surgery, as reported by Wells et al.^16^, has been shown to improve patient outcomes, underscoring the importance of postoperative management training and specialization. Advancements in surgical approaches, such as the use of three-dimensional (3D) models and augmented reality over conventional imaging for preoperative assessment in Wilms tumor cases^17,18^, also emphasize the impact of specialized training. Furthermore, fellowship in pediatric oncologic surgery has been linked to improved survival rates, indicating the positive effect of specialized training on optimizing therapy based on risk stratification algorithms^9^. The expertise and ongoing training of pediatric surgeons are also critical in managing surgical complications in pediatric oncology patients, requiring technical skills and a deep understanding of treatment guidelines and care specific to this population. Additionally, adapting standardized surgical approaches in resource-limited settings poses challenges, highlighting the importance of specialization and training in devising local control strategies to improve outcomes^13^.

The field of pediatric surgical oncology is undergoing a transformative phase, marked by rapid advancements in technology and surgical methods. Robotic surgery has added a new dimension to pediatric oncology, providing enhanced precision, dexterity, and superior visualization. This minimally invasive approach facilitates complex procedures, leading to reduced blood loss, improved cosmetic outcomes, and potentially better surgical margins. The use of robotic surgery in the resection of pediatric solid tumors has shown promising results in terms of patient recovery and postoperative quality of life^19^. Despite its advantages, the integration of robotic surgery faces significant challenges, notably the high costs of equipment and the specialized training required for surgeons. These challenges are particularly acute in low- and middle-income countries (LMICs), where limited resources and access to training can impede the widespread adoption of robotic surgery. Additionally, the learning curve associated with these techniques necessitates ongoing education and skill development among surgical teams^13,15,19^.

The advent of 3D modeling and augmented reality has revolutionized preoperative preparation in pediatric surgical oncology. These technologies offer an unparalleled depth of insight into complex anatomical structures, enabling surgeons to plan and execute surgical interventions with enhanced precision. 3D reconstructions, including cinematic rendering and volume rendering, provide a detailed visualization of pediatric tumors, significantly aiding in therapeutic decisions and prognostic assessments in various areas such as thoracic, brain, urology, and abdominal surgery^18^. The use of these technologies in the preoperative assessment of children, for instance, in cases like Wilms tumors, has demonstrated potential for guiding surgical decision-making^17^. These advanced imaging techniques enable surgeons to tailor diagnosis and treatment plans more accurately, thus improving surgical outcomes and patient care. However, the integration of these technologies in varied clinical settings, especially in low-resource environments, presents challenges, including the limited availability of advanced imaging equipment, the high costs involved, and the need for specialized training^17,18,20^.

Enhanced recovery after surgery (ERAS) programs in pediatric oncology represent a significant stride toward improving surgical precision, enhancing patient safety, and reducing postoperative complications. By leveraging advanced techniques like 3D imaging and augmented reality, these programs provide detailed anatomical visualizations that aid in meticulous surgical planning. The multidisciplinary nature of ERAS, involving collaboration among surgeons, anesthesiologists, nurses, and other healthcare professionals, is crucial in minimizing the physiological stress associated with surgery. Studies have indicated that ERAS protocols in pediatric oncology surgery lead to faster recovery, reduced hospital stays, and fewer complications, thereby improving the quality of life for pediatric patients^16^. Despite their benefits, the application of ERAS protocols, particularly in the surgical resection of solid tumors in children, requires further research, including randomized prospective studies. Moreover, implementing these protocols in resource-limited settings encounters hurdles such as limited resource availability, a lack of awareness, and resistance to change^21^.

Looking ahead, pediatric surgical oncology faces several challenges that need to be addressed to continue advancing the field. First, the disparities in the availability of advanced surgical techniques and technologies between high-income countries and LMICs present a major challenge^5,6,7,22^. Addressing these disparities requires a concerted effort to improve educational infrastructure, training programs, and access to essential resources globally. Second, the implementation of innovative surgical approaches such as 3D modeling, augmented reality, and robotic surgery in LMICs is hindered by financial constraints, a lack of infrastructure, and the need for specialized training. Collaborations between healthcare institutions, governments, and international organizations are essential to provide the necessary support and infrastructure development. Third, the ethical considerations in conducting research involving pediatric patients, obtaining funding for research projects, and ensuring long-term follow-up and data collection are challenges that researchers in pediatric surgical oncology face. Fostering a research environment that emphasizes ethical practices and long-term patient care is vital. Finally, keeping pace with rapid technological advancements while ensuring equitable access to these innovations remains a significant challenge. The field must balance the excitement of new technologies with the practicalities of their implementation in diverse healthcare settings.

## CONCLUSION

The landscape of pediatric surgical oncology is undergoing a transformative era, marked by remarkable technological advancements and strategic approaches that significantly impact the prognosis of childhood cancer. The integration of minimally invasive and robotic surgeries, alongside the innovative preoperative planning and recovery strategies embodied by ERAS programs, represents a leap forward in enhancing surgical precision, reducing patient morbidity, and accelerating recovery. However, the journey forward is not without challenges, particularly in harmonizing these advances across diverse global healthcare settings and ensuring equitable access to these cutting-edge technologies and methods.

As we look toward the future, the field of pediatric surgical oncology stands on the precipice of an exciting era of research and development. The continued collaboration, innovation, and commitment to overcoming the existing barriers will be pivotal in shaping a world where every child battling cancer has the best possible prognosis and quality of life, irrespective of their geographical or socioeconomic background. This journey, though complex, is filled with hope and potential, promising a brighter, healthier future for children worldwide facing the challenges of cancer.
